# Metabolic profile in endothelial cells of chronic thromboembolic pulmonary hypertension and pulmonary arterial hypertension

**DOI:** 10.1038/s41598-022-06238-z

**Published:** 2022-02-10

**Authors:** V. F. E. D. Smolders, C. Rodríguez, I. Blanco, R. Szulcek, Wim Timens, L. Piccari, Y. Roger, X. Hu, Constanza Morén, C. Bonjoch, L. Sebastián, M. Castellà, J. Osorio, V. I. Peinado, Harm Jan Bogaard, P. H. A. Quax, M. Cascante, J. A. Barberà, O. Tura-Ceide

**Affiliations:** 1 Department of Pulmonary Medicine, Servei de Pneumologia, Hospital Clínic-Institut d’Investigacions Biomèdiques August Pi i Sunyer (IDIBAPS), University of Barcelona, Villarroel, 170, 08036 Barcelona, Spain; 2 Department of Surgery, Leiden University Medical Center, Leiden, The Netherlands; 3 Biomedical Research Institute-IDIBGI, Girona, Spain; 4 Centro de Investigación Biomédica en Red (CIBER) de Enfermedades Respiratorias, Madrid, Spain; 5 Laboratory of in Vitro Modeling Systems of Pulmonary Diseases, Institute of Physiology, Charité – Universitätsmedizin Berlin, Corporate Member of Freie Universität Berlin and Humboldt-Universität zu Berlin, Charitéplatz, Berlin, Germany; 6 German Heart Center Berlin, Berlin, Germany; 7 Department of Pathology and Medical Biology, University of Groningen, University Medical Center Groningen, Groningen, the Netherlands; 8 Laboratory of Muscle Research and Mitochondrial Function, Institut d’Investigacions Biomèdiques August Pi i Sunyer (IDIBAPS), Department of Internal Medicine, Hospital Clínic of Barcelona (HCB), University of Barcelona (UB), Barcelona, Spain; 9 Biomedical Research Networking Centre on Rare Diseases (CIBERER), Madrid, Spain; 10 Department of Cardiovascular Surgery, Institut Clínic Cardiovascular, Hospital Clínic, Barcelona, Spain; 11 Amsterdam UMC, Department of Pulmonary Diseases, Amsterdam Cardiovascular Sciences (ACS), VU University Medical Center (VUmc), Amsterdam, the Netherlands; 12 Department of Biochemistry and Molecular Biomedicine and Institute of Biomedicine (IBUB), Faculty of Biology, Universitat de Barcelona, Diagonal 643, 08028 Barcelona, Spain; 13 CIBER of Hepatic and Digestive Diseases (CIBEREHD), Institute of Health Carlos III (ISCIII), 28029 Madrid, Spain

**Keywords:** Molecular biology, Cardiology, Medical research, Diseases, Cardiovascular diseases, Respiratory tract diseases, Cell biology, Mitochondria, Energy metabolism

## Abstract

Chronic thromboembolic pulmonary hypertension (CTEPH) and pulmonary arterial hypertension (PAH) are two forms of pulmonary hypertension (PH) characterized by obstructive vasculopathy. Endothelial dysfunction along with metabolic changes towards increased glycolysis are important in PAH pathophysiology. Less is known about such abnormalities in endothelial cells (ECs) from CTEPH patients. This study provides a systematic metabolic comparison of ECs derived from CTEPH and PAH patients. Metabolic gene expression was studied using qPCR in cultured CTEPH-EC and PAH-EC. Western blot analyses were done for HK2, LDHA, PDHA1, PDK and G6PD. Basal viability of CTEPH-EC and PAH-EC with the incubation with metabolic inhibitors was measured using colorimetric viability assays. Human pulmonary artery endothelial cells (HPAEC) were used as healthy controls. Whereas PAH-EC showed significant higher mRNA levels of GLUT1, HK2, LDHA, PDHA1 and GLUD1 metabolic enzymes compared to HPAEC, CTEPH-EC did not. Oxidative phosphorylation associated proteins had an increased expression in PAH-EC compared to CTEPH-EC and HPAEC. PAH-EC, CTEPH-EC and HPAEC presented similar HOXD macrovascular gene expression. Metabolic inhibitors showed a dose-dependent reduction in viability in all three groups, predominantly in PAH-EC. A different metabolic profile is present in CTEPH-EC compared to PAH-EC and suggests differences in molecular mechanisms important in the disease pathology and treatment.

## Introduction

Pulmonary hypertension (PH) is defined as an increase in mean pulmonary arterial pressure (mPAP) > 20 mmHg^1^. Based on similar pathogenesis, clinical and haemodynamics characteristics, PH is categorized into five clinical groups: pulmonary arterial hypertension (PAH); PH due to left heart disease; PH associated with lung disease and/or hypoxia; chronic thromboembolic pulmonary hypertension (CTEPH) and PH with unclear or multifactorial mechanisms^2^. Although the most common cause of PH is left heart disease, PAH and CTEPH have received the largest interest of researchers over the years^2,3^. PAH is a fatal disease characterized by a sustained increase in pulmonary vascular resistance mainly due to microvascular remodeling that eventually may lead to right ventricular failure and death^1,2^. The increase in pulmonary vascular resistance is due to vessel wall remodeling in pre-capillary arteries and arterioles characterized by thickening of the endothelial and ∕or smooth muscle layer and by the presence of plexiform lesions^4–6^. CTEPH is a severe clinical form of PH^1^, defined by increased mPAP due to non-resolved thrombotic lesions in pulmonary arteries despite appropriate anticoagulant therapy^1^. To date, exact molecular and metabolic mechanisms, similarities, and divergences between PAH and CTEPH and regulatory pathways behind vascular remodeling and disease progression are not fully resolved.

The pulmonary vascular endothelium plays key roles in maintaining vessel homeostasis^7^. Under physiological conditions the endothelium maintains a quiescent state with a predominant use of glycolysis, fatty acids, and amino acids as their main sources of energy and biosynthetic precursors^8^ rather than oxidative phosphorylation in the mitochondria. The glycolytic pathway is paralleled by the pentose phosphate pathway (PPP) that uses glycolytic intermediates, mediated through glucose-6-phosphate-dehydrogenase (G6PD), but also shunts intermediates back into the glycolysis pathway when needed^8^. Even though endothelial cells (ECs) mainly rely on glycolysis for adenosine triphosphate (ATP) production, mitochondria remain fully functional and rather works as suppliers for cellular building blocks through metabolism of amino acids such as glutamine and glutamate that serve as biosynthetic precursors to produce nucleotides and macromolecules through glutaminolysis^9^.

Upon endothelial stimulation or injury, the endothelium switches to an activated state which is associated with changes in cellular metabolism, EC dysfunction and pulmonary vascular remodeling^10,11^. Under sustained pathological conditions, EC metabolic alterations promote vascular diseases by means of excessive cellular proliferation, increased angiogenesis, and a pro-survival cellular phenotype^8,10^. ECs isolated from vascular lesions in patients with PAH are found to have a hyperproliferative and apoptosis-resistant phenotype that is supported by a metabolic switch towards glycolysis, changes in oxidative phosphorylation and increased glutaminolysis^12–14^. Increased expression of pyruvate dehydrogenase kinase (PDK) in PAH is thought to be responsible for the augmented reliance on glycolysis^15,16^. Additionally, Bujak et al*.,* found in PAH a clear imbalance of certain metabolites related to energy homeostasis such as glycolysis and lipid and amino acid associated metabolites^17^. To date, it is unknown whether pulmonary artery ECs from CTEPH patients share the same metabolic characteristics as pulmonary artery ECs from PAH or whether a metabolic modulation might be beneficial in patients with CTEPH. We have recently demonstrated that ECs isolated from CTEPH patients have a reduced glycolysis compared to control endothelial cells^18^. Additionally, Heresi et al., using plasma samples instead of ECs, identified significant unique metabolomic differences between age and gender matched PAH and CTEPH samples namely, increased lipolysis and altered ammino acid metabolism^19^.

In this study, we isolated ECs from vascular tissue collected at pulmonary endarterectomy (PEA) and after lung transplantation from CTEPH and PAH patients, respectively and we perform a systematic metabolic comparison of ECs from CTEPH and PAH patients. To distinguish between the microvascular and macrovascular origin of ECs isolated from the CTEPH patients, we also analyzed homeobox D cluster (HOXD) expression on these cells as it has been shown that the expression patterns of the HOXD gene family, and especially HOXD3, -8 and -9, allow to distinguish between the microvascular and macrovascular origin of ECs^20^. In addition, we assessed, basal viability and viability upon inhibition by metabolic regulators of glycolysis and glutaminolysis and endothelial identity based on previous studies.

## Materials and methods

### Study population and samples collected

This study included ECs from twelve CTEPH patients who underwent pulmonary endarterectomy (PEA) at the Hospital Clinic of Barcelona, Spain. This study further included six patients with end-stage PAH who underwent lung transplantation at University Medical Center Groningen, Dept. of Pathology and Medical Biology, Groningen, the Netherlands followed by ECs isolation performed at the Amsterdam UMC, VU University Medical Center (VUmc), Dept. of Pulmonary Diseases, Amsterdam Cardiovascular Sciences (ACS), Amsterdam, the Netherlands. The study was conducted in accordance with the Declaration of Helsinki, and it was approved by the institutional Ethics Committee on Human Research (Hospital Clínic of Barcelona ethics committee (HCB/2018/0837 and HCB/2018/0434) of the Hospital Clínic of Barcelona and the institutional review board (IRB) for human studies of the Amsterdam UMC at VU University Medical Center (VUmc, Amsterdam, the Netherlands) (non-WMO, 2012/306). Written informed consent was obtained from the subjects or their surrogates for the collection of materials and publication of results, if required. The patients received.

PH-targeted therapy either as treatment of the disease (PAH), or as a bridge to the intervention (CTEPH). In PAH patients’ combination therapy prevailed with endothelin antagonists (ERAs) together with phosphodiesterase type 5 inhibitors (PDE5i) and/or plus prostanoids, whereas in CTEPH patients received either monotherapy (ERAs/PDE5i/Riociguat), combination therapy (ERA plus PDE5i or Riociguat) or no therapy.

### Pulmonary endothelial cell isolation and culture

ECs isolated from endarterectomy specimens of CTEPH patients, referred to as CTEPH-EC*,* were isolated and cultured as previously described^18^. EC phenotype was characterized by positive staining of endothelial and smooth muscle cell-specific markers, including endothelial nitric oxide synthase (eNOS) and alpha-smooth muscle actin (α-SMA) as previously described^21^. ECs from two heritable and four idiopathic PAH patients (referred to as PAH-EC) were isolated from lung tissues and artery rings of end-stage PAH patients obtained from lung transplantations and cultured as previously described^22,23^. ECs were purified by magnetic affinity cell sorting (MACS, Miltenyi Biotec) based on CD144 antibody labeling and purity was ensured by regular FACS testing. In short, PAECs were plated onto 0.2% gelatin-coated wells and grown in endothelium cell growth medium-2 (EGM-2) supplemented with EGM-2 SingleQuots (Lonza, USA) and 10% fetal bovine serum (FBS) (GE Healthcare, USA). Patients’ characteristics are presented in Table 1. Human pulmonary artery endothelial cells (HPAEC) (Lonza, CC-2530) were used as control cells in all experiments. Human lung microvascular endothelial cells (HMVEC-L) were used as positive controls only in HOXD gene expression studies.

**Table 1:** Clinical features and hemodynamic parameters.

	CTEPH (n = 12)	PAH (n = 6)
Female/male	6/6	5/1
Age years	58.0 ± 7.6	37.1 ± 10.7*
BMI kg·m^−2^	26.6 ± 4.0	27.8 ± 16.5
mPAP mmHg	40.6 ± 8.8	68.1 ± 24.2*
PVR dyn·s·m^−5^	607 ± 246	1070 ± 464 ^p=0.05^
PAOP mmHg	9.7 ± 4.4	12.3 ± 6.6
Cardiac index L·min^−1^·m^−2^	2.3 ± 0.4	2.56 ± 0.8
Right atrial pressure mmHg	7.0 ± 5.2	13.83 ± 7.3
SvO2%	61.8 ± 7.6	52.00 ± 4.9 ^p=0.07^
BNP pg·mL^−1^	166 ± 300	6021 ± 4939*
**WHO FC**^**b**^		
I	0	0
II	3	0
III	9	3
IV	0	1

Data are presented as n or mean ± SD.

*CTEPH* chronic thromboembolic pulmonary hypertension, *PAH* pulmonary arterial hypertension, *BMI* body mass index, *mPAP* mean pulmonary artery pressure, *PVR* pulmonary vascular resistance, *PAOP* pulmonary artery occlusion pressure, *SvO2* mixed venous oxygen blood saturation, *BNP* brain natriuretic peptide, *WHO FC* world health organization functional class.

*p < 0.05, unpaired t-test, data expressed as mean ± SD.

### Gene expression analyses

The mRNA levels of metabolic genes were measured by qPCR (n = 10 CTEPH-EC, n = 6 PAH-EC and n = 6 HPAEC). ECs were seeded at a density of 6 × 10^4^ per 40 mm cell culture dish (pre-coated with 0.2% gelatin). Total RNA was extracted using TRIsure (Bioline, Germany) and concentrations were determined by spectrophotometry. Reverse transcription was performed using reactive mix high-Capacity cDNA RT kit (Applied Biosystems, USA). For qRT-PCR, SYBR Green I (ThermoFisher Scientific, USA) and specific primers were used on the ViiA7 Real-Time PCR system (Applied Biosystems, USA). Relative quantification was calculated by normalizing the Ct (threshold cycle) of the gene of interest to the Ct of an endogenous control (β-actin) in the same sample, using the comparative ΔΔCt method. All primers were designed with Primer 3 Plus and delivered by Integrated DNA Technologies. Primer sequences can be found in supplementary Table 1.

### Protein expression analyses

ECs from n = 6 CTEPH, n = 5 PAH patients and n = 3 HPAEC were seeded into T75 flasks pre-coated with 0.2% gelatin, at a density of 4 × 10^5^ cells per flask and allowed to adhere and grow in complete endothelial medium till confluency was reached. Next, cells were washed twice with ice-cold PBS and treated with Pierce RIPA buffer (ThermoFisher Scientific, USA) supplemented with a Halt protease/phosphatase inhibitor cocktail (ThermoFisher Scientific, USA). After incubation on ice, the cell lysate was recovered and centrifugated. Protein concentrations in the lysates were determined using the Pierce BCA protein assay kit (ThermoFisher Scientific, USA). Then, 15-25 μg of total protein was loaded onto 4–12% Bis–Tris Gels (ThermoFisher Scientific, USA). The proteins were transferred to nitrocellulose membranes and blocked for 1 h at room temperature with 1X blocking solution using Casein Blocking Solution 10X (Sigma-Aldrich, USA). Membranes were incubated overnight at 4ºC under rotation in 0.5X Casein Blocking Solution with primary antibodies following the manufacturer recommendations. Horseradish peroxidase-conjugated anti-IgG was used as the secondary antibody. Immunoreactive bands were detected by WesternBright Quantum substrate (Advansta, USA). Images were analyzed with freely available imaging processing ImageJ software, version:2.1.0/1.53c, http://imagej.net/contributors. The antibodies list can be found in supplementary Table 2.

### Cell viability assay

ECs from n = 3 CTEPH donors, n = 3 PAH donors and n = 4 HPAEC were seeded into 96-wells pre-coated with 0.2% gelatin, at a density of 5 × 10^3^ cells per well and allowed to adhere and grow in 10% FBS EGM-2 medium till 80% confluency was reached. Inhibition treatments were performed in starving medium (0.5% FBS EGM-2) and incubated at 37 °C for 24 h. 3PO (15 and 30 μM), BPTES (2 and 4 μM), UK-5099 (2 and 4 μM) and DCA (40 and 80 mM) were used to evaluate the effects on cell viability by blocking different metabolic pathways. The Vybrant MTT Cell Proliferation Assay Kit (ThermoFisher Scientific, USA) was used to determine cellular viability. Medium was initially exchanged for 0.5% FBS EGM-2 phenol-red-free medium with or without inhibitors depending on the experimental conditions. Basal viability was measured in 10% FBS EGM-2 phenol-red-free medium. 20 mM MTT solution was added and incubated for four hours at 37 °C. SDS 0.01 M HCl solution was then loaded followed by an additional four-hour incubation at 37 °C. The absorbance was recorded at 570 nm on Synergy HTX Multi-Mode Microplate Reader.

### Statistical analysis

Results are shown as means ± standard deviation. Comparisons were performed with One Way Anova for normally distributed variables or Kruskal–Wallis One Way Analysis of Variance and when significant, post-hoc Dunn’s multiple comparisons test for non-normally distributed variables. Cellular viability analyses were performed using Two-way ANOVA followed by Tukey´s multiple comparison test. Statistical analyses were performed using GraphPad Prism 7 software, version 7.0e, serial number:GP7-0633739-R###-#####, https://www.graphpad.com. *p* values ≤ 0.05 were considered statistically significant.

### Ethics approval and consent to participate

All procedures performed in studies involving human participants were in accordance with the ethical standards of the institutional and/or national research committee and with the 1964 Helsinki Declaration and its later amendments or comparable ethical standards. The study protocol was granted by the Hospital Clínic of Barcelona ethics committee (HCB/2018/0837 and HCB/2018/0434).

### Consent for publication

Informed consent was obtained from all subjects involved in the study.

## Results

### Clinical data

Clinical data are summarized in Table 1. ECs were isolated from a total of eighteen PH patients, twelve patients with CTEPH and six with PAH. Generally, patients with PAH were mostly female, younger, and had more severe hemodynamic impairment than patients with CTEPH. All PAH and eight CTEPH patients were treated with PAH targeted therapy. The other four CTEPH patients did not receive any PAH specific treatment.

### HOXD expression

**Figure 1:**
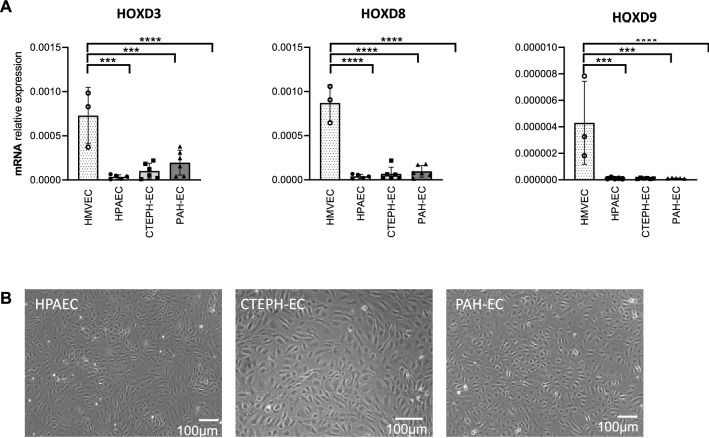
HOXD gene expression. (**A**) mRNA expression levels of HOXD3, HOXD8 and HOXD9 were found significantly higher in HMVEC-L compared to HPAEC, CTEPH-EC and PAH-EC. No differences in gene expression were found between HPAEC, CTEPH-EC and PAH-EC. HMVEC-L, n = 3; HPAEC, n = 5; CTEPH-EC, n = 6; PAH-EC, n = 3; One-way ANOVA, *p* < 0.001 = ***; *p* < 0.0001 = ****, data is expressed as mean ± SD. Statistical analysis was performed with GraphPad Prism 7 software, version 7.0e, serial number:GP7-0633739-R###-#####,https://www.graphpad.com. (**B**) Representative pictures of cultured HPAEC, CTEPH-EC and PAH-EC, × 10 magnification.

HOXD gene expression in PAH-EC, CTEPH-EC and HPAEC was similar among them and significantly lower compared to HMVEC-L, used as microvascular positive control (Fig. 1A). Figure 1B shows a representative image of PAH-EC, CTEPH-EC and HPAEC subject groups (Fig. 1B).

### Metabolic gene expression

**Figure 2:**
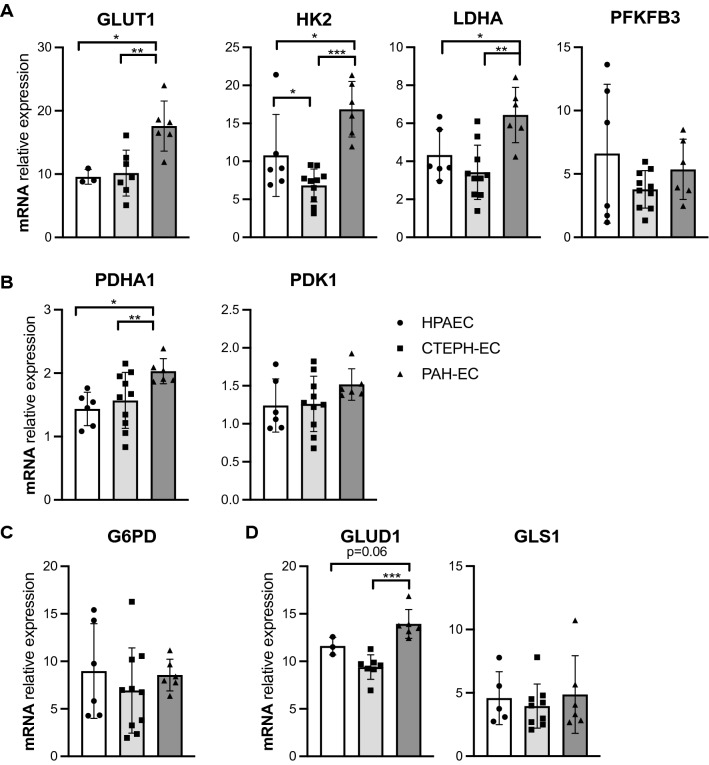
Gene expression of key metabolic enzymes. (**A**) mRNA expression levels of GLUT1, HK2, LDHA and PFKFB3 in CTEPH-EC, PAH-EC and HPAEC. (**B**) mRNA levels of PDHA1, PDK1 in all three groups. (**C**) G6PD mRNA levels were found similar between all three groups. (**D**) mRNA levels of metabolic enzyme GLS1 and GLUD1 in all three groups studied. CTEPH-EC, n > 7; PAH-EC, n = 6; HPAEC, n > 3; One-way ANOVA, data is expressed as mean ± SD, *p* < 0.05 = *; *p* < 0.01 = **; *p* < 0.001 = ***. Statistical analysis was performed with GraphPad Prism 7 software, version 7.0e, serial number:GP7-0,633,739-R###-#####,https://www.graphpad.com.

PAH-EC showed a significant increased gene expression of glycolytic enzymes glucose transporter 1 (GLUT1), hexokinase 2 (HK2) and lactate dehydrogenase A (LDHA) compared with control cells and CTEPH-EC (Fig. 2A). Glycolytic regulator 6-phosphofructo-2-kinase/fructose-2,6-biphosphatase 3 (PFKFB3) did not show any difference at mRNA level between the three subject groups (Fig. 2A). Mitochondrial gate-keeper enzyme pyruvate dehydrogenase subunit E1 alpha (PDHA1) also presented higher expression of mRNA levels in PAH-EC than control cells and CTEPH-EC (Fig. 2B), while mRNA levels of PDK1 were not found differently expressed between all three groups (Fig. 2B). Additionally, the three groups expressed similar mRNA levels of PPP associated G6PD (Fig. 2C). Although glutamate converting enzyme glutamate dehydrogenase 1 (GLUD1) presented higher mRNA levels in PAH-EC, glutamine converting enzyme, glutaminase 1 (GLS1) expression was similar in all three PAH-EC, CTEPH-EC and HPAEC (Fig. 2D).

### Metabolic protein expression

**Figure 3:**
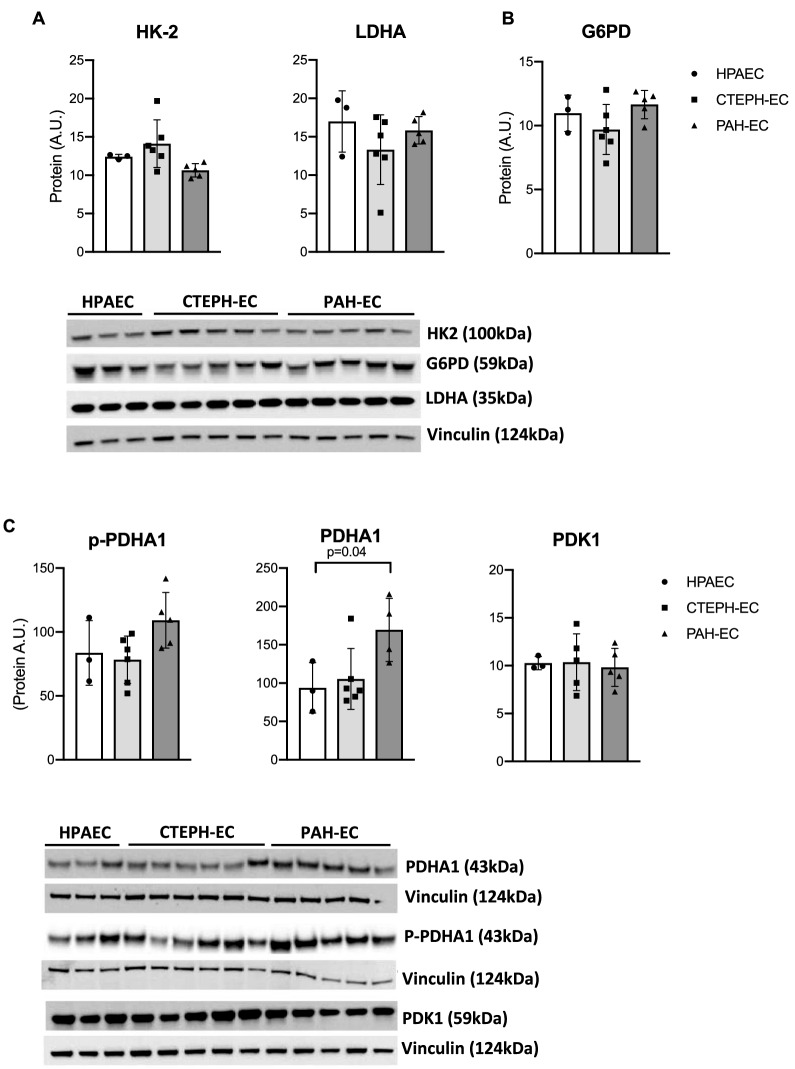
Protein expression of key metabolic enzymes. (**A**) Protein levels of HK2 and LDHA in CTEPH-EC, PAH-EC and HPAEC. (**B**) Protein levels of PPP key enzyme G6PD were similar in all three groups studied. (**C**) Protein levels of the phosphorylated E1 alpha PDH subunit (p-PDHA-1), PDHA1 (non-phosphorylated subunit) and PDHA1 inhibitor, PDK1 in all three groups. CTEPH-EC, n = 5–6; PAH-EC, n = 5; HPAEC, n = 3; Kruskal–Wallis test followed by Dunn´s multiple comparisons test, *p* < 0.05 = *; data is expressed as mean ± SD. Statistical analysis was performed with GraphPad Prism 7 software, version 7.0e, serial number:GP7-0,633,739-R###-#####, https://www.graphpad.com. (full-length gels are presented in supplementary material Fig. 1).

Protein levels of glycolytic rate-limiting enzymes HK2 and LDHA were found similarly expressed between all three study groups (Fig. 3A). G6PD protein expression were also not different between CTEPH-EC, PAH-EC and HPAEC (Fig. 3B). However, protein levels of the phosphorylated and non-phosphorylated PDHA1 showed a tendency of higher expression in PAH-EC towards CTEPH-EC and HPAEC reaching statistical significance in PDHA1 between PAH-EC compared to HPAEC (Fig. 3C). No difference was observed in protein levels of PDK1 between the three groups (Fig. 3C).

**Figure 4:**
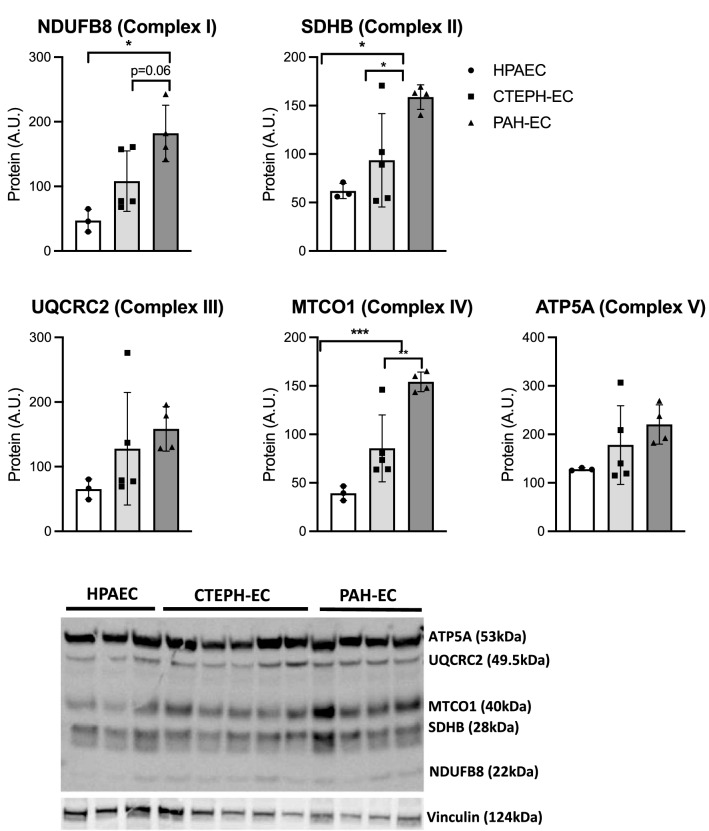
Oxidative associated protein expression. Protein levels of complexes-I, -II and -IV were found significantly upregulated in PAH-EC compared to CTEPH-EC. All three complexes are also significantly different between PAH-EC and HPAEC. Similar levels were observed for complexes-III and -V between all three groups studied. CTEPH-EC, n = 5; PAH-EC, n = 4; HPAEC, n = 3; One-way ANOVA, data is expressed as mean ± SD, *p* < 0.05 = *; *p* < 0.01 = **; *p* < 0.001 = ***. Statistical analysis was performed with GraphPad Prism 7 software, version 7.0e, serial number:GP7-0,633,739-R###-#####,https://www.graphpad.com. (full-length gels are presented in supplementary material Fig. 2).

Additionally, oxidative phosphorylation associated proteins from complex-I, complex-II and complex-IV were studied and presented an increased expression in PAH-EC compared to CTEPH-EC and HPAEC (Fig. 4). Protein levels of complexes-III and -V showed a similar expression between CTEPH-EC and HPAEC (Fig. 4).

### Cellular viability

**Figure 5:**
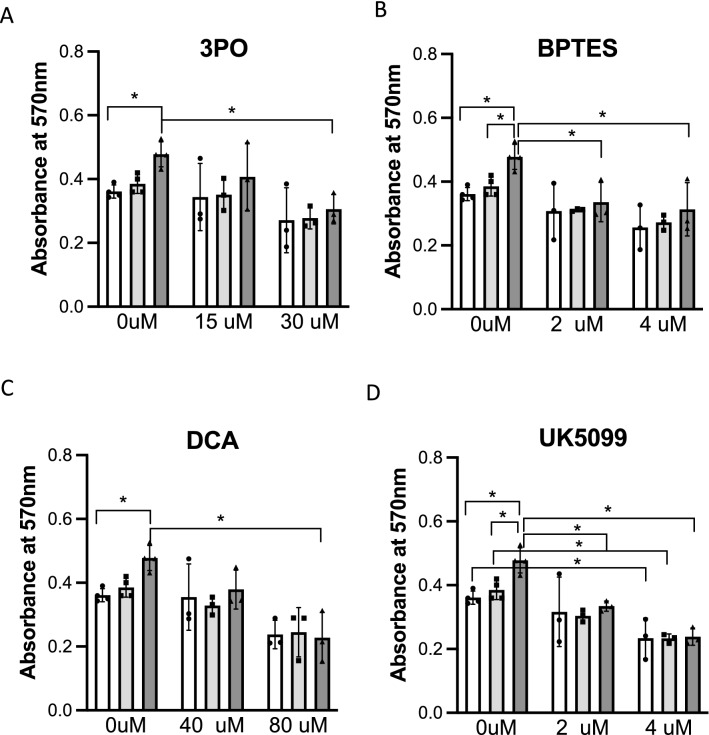
Cellular basal viability and upon metabolic inhibition. Cellular basal viability and the effect of metabolic inhibition was evaluated by use of MTT. 3PO (glycolytic inhibitor), BPTES (glutaminase inhibitor), UK5099 (mitochondrial pyruvate carrier blocker) and DCA (PDK1 inhibitor) showed a dose dependent effect on the viability of all three groups studied. CTEPH-EC, n = 3; PAH-EC, n = 3; HPAEC, n = 4; Two-way ANOVA followed by Tukey´s multiple comparison test was used. Data is expressed as mean ± SD, *p* < 0.05 = *. Statistical analysis was performed with GraphPad Prism 7 software, version 7.0e, serial number:GP7-0,633,739-R###-#####,https://www.graphpad.com.

Due to the clear metabolic differences observed between CTEPH-EC and PAH-EC, basal viability was assessed to investigate a relationship between the observed changes in cell metabolism and cellular functions. Basal viability was higher in PAH-EC compared to HPAEC and CTEPH-EC (Fig. 5). To determine functional impact of metabolic changes, cellular viability after incubation with metabolic inhibitors was assessed in all three groups. Incubation with glycolytic inhibitor 3-(3-pyridinyl)-1-(4-pyridinyl)-2-propen-1-one (3PO) showed a dose-dependent reduction in viability in all three groups reaching statistical significance between PAH-EC grown at basal conditions compared to PAH-EC incubated with 30uM 3PO (Fig. 5A). Likewise, for glutaminase inhibitor bis-2-(5-phenylacetamido-1,3,4-thiadiazol-2-yl)-ethyl-sulfide (BPTES), and PDK inhibitor dichloroacetate (DCA) a dose-dependent reduction in residual viability was observed in all three groups being significantly affected in PAH-EC (Fig. 5B,C) Mitochondrial pyruvate carrier blocker 2-Cyano-3-(1-phenyl-1H-indol-3-yl)-2-propenoic-acid (UK-5099) showed a significant dose-dependent reduction in viability in all three groups but no difference was found in viability among groups (Fig. 5D).

## Discussion

This study showed a significant difference in the reliance on glycolysis between CTEPH-EC and PAH-EC. PAH-EC presented higher expression levels of glycolytic and glutamine-related enzymes compared to CTEPH-EC and healthy ECs. The increase in glycolytic enzymes in PAH-EC compared to CTEPH-EC was further accompanied by an increase in PDHA1 mRNA levels. Furthermore, protein levels of oxidative phosphorylation complexes I, II and IV were higher in PAH-EC compared to CTEPH-EC. Accordingly, a substantially different metabolic profile at the level of glycolysis, oxidative phosphorylation and glutamine metabolism is present in PAH-EC compared to CTEPH-EC and suggests differences in molecular mechanisms and regulatory pathways that could be important in the disease pathology and treatment.

In this study, a similar HOXD gene expression pattern between CTEPH-EC and PAH-EC was shown. Toshner et al*.*^20^ described that, considering the HOXD expression patterns, ECs could be clustered based on the type of blood vessel that they were derived from. Their study showed that high expression of HOXD3, HOXD8 and HOXD9 was associated with ECs that are microvascular in origin^20^. In the present study, the expression profiles of all 3 HOXD genes, both in CTEPH-EC and PAH-EC, were similar to HPAEC. Based on this data it can be concluded that CTEPH-EC and PAH-EC used in the present study origin both from a macrovascular lineage.

It is known that, in PAH, vascular ECs adopt metabolic changes associated with vascular hyperproliferation and resistance to apoptosis. PAH-EC are found to increase glycolysis in order to assure proliferation^13,24^. Although previous studies in CTEPH have shown an hyperproliferative dysfunctional phenotype in CTEPH endothelial cells^25,26^, not much is known about metabolic alterations in CTEPH-EC. Based on recent in vitro and in vivo studies from our group, CTEPH-EC seem to be associated with impairments in their metabolism that point towards lower glycolysis and glutaminolysis in CTEPH-EC compared to healthy ECs^18,27^. In accordance with those observations, the present study showed the upregulation of glycolytic genes GLUT1, HK2 and LDHA in PAH-EC compared to CTEPH-EC indicating different metabolic profiles between both PH diseases. Glycolytic regulator PFKFB3 was not found differently expressed between all EC types studied. An explanation could be that PFKFB3 activity is dependent mainly on post-translational modifications^28,29^.

Another important key feature of metabolic adaptations in ECs from PAH patients found in this study, is the increased expression of PDHA1 compared to CTEPH-EC and healthy ECs. PDK1 phosphorylates PDHA1, which blocks mitochondrial oxidative phosphorylation and further promotes glycolysis^30,31^. The present study showed increased gene and protein expression of PDHA1 in PAH-EC compared to CTEPH-EC and healthy ECs, but no difference in gene or protein expression of PDK1. Contrary to previous results^16^ and regardless an increased glycolysis, we could not find an upregulation of PDK1 in PAH-EC or a downregulation of mitochondrial activity. Protein levels of oxidative phosphorylation complexes I, II and IV were increased in PAH-EC compared to CTEPH-EC and healthy ECs indicative for an elevated functional mitochondrial respiration and could explain the increase in active PDHA1 which is in line with the increased use of glycolysis.

Besides glycolysis and oxidative phosphorylation, glutamine metabolism is thought to be involved in PAH pathology and has been shown to be altered in CTEPH-EC^18,32^. Glutamine metabolism is essential in EC proliferation and is driven by the expression of GLS1 and GLUD1^9^. The current study showed increased gene expression of GLUD1 but not GLS1 in PAH-EC compared to CTEPH-EC. This observation implies a role of glutamine metabolism in PAH-EC but, also, further confirms a difference in metabolism between PAH-EC and CTEPH-EC that needs deeper attention. At last, the oxidative arm of the PPP, important for maintaining cell viability under high rates of proliferation^33^, was not found different between CTEPH-EC and PAH-EC, neither between PAH-EC and healthy ECs.

Based on the EC metabolic and viability profile between CTEPH and PAH, ECs were treated with metabolic inhibitors to understand whether differences in metabolism could be translated into differences in viability upon inhibition. All ECs studied showed a dose-dependent reduction in viability after incubation with metabolic inhibitors 3PO, DCA, BPTES and UK-5099. Particularly, PAH-EC were further affected when cultured with 3PO, DCA, BPTES compared to HPAEC and CTEPH-EC. These results are in line with the higher expression level of glycolytic and glutamine-related enzymes found in PAH-EC compared to CTEPH-EC and healthy ECs. Previous studies have shown beneficial effects of orphan small molecule DCA in both human PAH and experimental PAH^15,16^. DCA is a pyruvate analogue which inhibits all four PDK isoforms and activates PDH activity. Although there was a dose response in CTEPH-EC when cultured with DCA, it did not reach statistically significance and suggest that DCA treatment may be better suited in PAH patients than CTEPH. UK-5099, is a potent inhibitor that blocks pyruvate transport into mitochondria. This inhibitor affected all cell lines without a clear difference among groups. On the other hand, it has been recently demonstrated that 3PO does not directly inhibit the enzymatic activity of key glycolytic enzymes such as HK, LDHA or PFKFB3^34^. Further studies need to test other glycolytic inhibitors to confirm these results.

Based on the results of this study, showing an endogenous glycolic difference in CTEPH-EC compared to PAH-EC, blocking glycolysis may not be beneficial in CTEPH-EC and requires further investigation for the development of novel CTEPH treatments.

This study presents some limitations. PAH patients were significantly younger and mostly female compared to CTEPH patients which is in line with the observed female predominance of PAH^35^ and the earlier onset of disease^36^*.* The significant increase in hemodynamic parameters in PAH patients compared to CTEPH patients could be explained by the more severe disease state of PAH patients included in this study. Differences in disease severity could potentially contribute to the differences in metabolism and viability between endothelial cells isolated from PAH and CTEPH patients. Additionally, our sample size is limited, and these results should be verified in a bigger patient group. Future work will aim to expand in PAH-EC, our recent results in oxygen consumption and mitochondrial functionality in CTEPH-EC, where we found an increased mitochondrial respiration activity in all the respiratory chain complexes compared to HPAEC^37^. Additionally*,* we aim to study the metabolism of pulmonary artery smooth muscle cells (PASMCs) of both diseases.

## Conclusion

Overall, our study demonstrates a novel different metabolic profile at the level of glycolysis, oxidative phosphorylation and glutamine metabolism in CTEPH-EC compared with PAH-EC (Table 2). This suggests differences in molecular mechanisms and regulatory pathways that could be important in disease pathology and in the development of curative treatments. More studies are needed to better understand the importance of reduced glycolysis and glutaminolysis in CTEPH-EC, and whether such differences may lead to the development of novel therapeutic approaches to treat CTEPH.

**Table 2:** Summary of the most relevant findings of CTEPH-EC in comparison with PAH-EC.

	CTEPH-EC
HOXD (mRBNA)	=
Glycolysis (mRNA)	**↓**
PDHA1 (mRNA)	**↓**
Glutaminolysis (mRNA)	**↓**
Oxidative phosphorylation complexes (protein)	**↓**
Basal viability	**↓**
Viability upon 3PO, BPTES and DCA treatment	**↑**

## Supplementary Information

Supplementary Information.

## Data Availability

All data relevant to the study are included in the article or uploaded as supplementary information.

## Abbreviations

ATP: Adenosine triphosphate
α-SMA: Alpha-smooth muscle actin
BPTES: Bis-2-(5-phenylacetamido-1,3,4-thiadiazol-2-yl)-ethyl-sulfide
Ct: Threshold cycle
CTEPH: Chronic thromboembolic pulmonary hypertension
CTEPH-EC: Chronic thromboembolic pulmonary hypertension endothelial cells
DCA: Dichloroacetate
ECs: Endothelial cells
eNOS: Endothelial nitric oxide synthase
FBS: Fetal bovine serum
GLS1: Glutaminase 1
GLUD1: Glutamate-dehydrogenase-1
GLUT1: Glucose transporter 1
G6PD: Glucose-6-phosphate-dehydrogenase
HK2: Hexokinase 2
HMVEC-L: Human lung microvascular endothelial cells
HOXD: Homeobox D cluster
HPAEC: Human pulmonary artery endothelial cells
LDHA: Lactate dehydrogenase A
mPAP: Mean pulmonary arterial pressure
PAH: Pulmonary arterial hypertension
PAH-EC: Pulmonary arterial hypertension endothelial cells
PDK: Pyruvate dehydrogenase kinase
PDHA1: Pyruvate dehydrogenase subunit E1 alpha
PEA: Pulmonary endarterectomy
PFKFB3: 6-Phosphofructo-2-kinase/fructose-2,6-biphosphatase 3
PH: Pulmonary hypertension
PPP: Pentose phosphate pathway
UK-5099: 2-Cyano-3-(1-phenyl-1H-indol-3-yl)-2-propenoic-acid
3-PO: 3-(3-Pyridinyl)-1-(4-pyridinyl)-2-propen-1-one

## References

[1] Simonneau G (2019). Haemodynamic definitions and updated clinical classification of pulmonary hypertension. Eur. Respir. J..

[2] Hoeper MM (2016). A global view of pulmonary hypertension. Lancet Respir. Med..

[3] Mann GMFaDL (2020). Heart Failure: A Companion to Braunwald's Heart Disease.

[4] Pietra GG (2004). Pathologic assessment of vasculopathies in pulmonary hypertension. J. Am. Coll. Cardiol..

[5] Humbert M (2010). Pulmonary arterial hypertension and chronic thromboembolic pulmonary hypertension: pathophysiology. Eur. Respir. Rev..

[6] Simonneau G, Torbicki A, Dorfmuller P, Kim N (2017). The pathophysiology of chronic thromboembolic pulmonary hypertension. Eur. Respir. Rev..

[7] Huertas A (2018). Pulmonary vascular endothelium: the orchestra conductor in respiratory diseases: Highlights from basic research to therapy. Eur. Respir. J..

[8] Eelen G (2018). Endothelial cell metabolism. Physiol. Rev..

[9] Kim B, Li J, Jang C, Arany Z (2017). Glutamine fuels proliferation but not migration of endothelial cells. EMBO J..

[10] Rohlenova K, Veys K, Miranda-Santos I, De Bock K, Carmeliet P (2018). Endothelial cell metabolism in health and disease. Trends Cell Biol..

[11] Budhiraja R, Tuder RM, Hassoun PM (2004). Endothelial dysfunction in pulmonary hypertension. Circulation.

[12] Masri FA (2007). Hyperproliferative apoptosis-resistant endothelial cells in idiopathic pulmonary arterial hypertension. Am. J. Physiol. Lung Cell. Mol. Physiol..

[13] Xu W (2007). Alterations of cellular bioenergetics in pulmonary artery endothelial cells. Proc. Natl. Acad. Sci. USA.

[14] Bertero T (2016). Vascular stiffness mechanoactivates YAP/TAZ-dependent glutaminolysis to drive pulmonary hypertension. J. Clin. Investig..

[15] Michelakis ED (2002). Dichloroacetate, a metabolic modulator, prevents and reverses chronic hypoxic pulmonary hypertension in rats: role of increased expression and activity of voltage-gated potassium channels. Circulation.

[16] Michelakis ED (2017). Inhibition of pyruvate dehydrogenase kinase improves pulmonary arterial hypertension in genetically susceptible patients. Sci. Transl. Med..

[17] Bujak R, Mateo J, Blanco I, Izquierdo-García JL, Dudzik D, Markuszewski MJ, Peinado VI, Laclaustra M, Barberá JA, Barbas C, Ruiz-Cabello J (2016). New biochemical insights into the mechanisms of pulmonary arterial hypertension in humans. PLoS ONE.

[18] Smolders VFED, Rodríguez C, Morén C, Blanco I, Osorio J, Piccari L, Bonjoch C, Quax PHA, Peinado VI, Castellà M, Barberà JA, Cascante M, Tura-Ceide O (2020). Decreased glycolysis as metabolic fingerprint of endothelial cells in chronic thromboembolic pulmonary hypertension. Am. J. Respir. Cell Mol. Biol..

[19] Heresi GA, Mey JT, Bartholomew JR, Haddadin IS, Tonelli AR, Dweik RA, Kirwan JP, Kalhan SC (2020). Plasma metabolomic profile in chronic thromboembolic pulmonary hypertension. Pulm. Circ..

[20] Toshner M (2014). Transcript analysis reveals a specific HOX signature associated with positional identity of human endothelial cells. PLoS ONE.

[21] Tura-Ceide O (2014). Derivation and characterisation of endothelial cells from patients with chronic thromboembolic pulmonary hypertension. Eur. Respir. J..

[22] Szulcek R (2016). Delayed microvascular shear adaptation in pulmonary arterial hypertension. Role of platelet endothelial cell adhesion molecule-1 cleavage. Am. J. Respir. Crit. Care Med..

[23] Manz XD, Albers HJ, Symersky P, Aman J, van der Meer AD, Bogaard HJ, Szulcek R (2020). In vitro microfluidic disease model to study whole blood-endothelial interactions and blood clot dynamics in real-time. J. Vis. Exp..

[24] Archer SL (2017). Pyruvate kinase and Warburg metabolism in pulmonary arterial hypertension: Uncoupled glycolysis and the cancer-like phenotype of pulmonary arterial hypertension. Circulation.

[25] Quarck R (2012). Characterization of proximal pulmonary arterial cells from chronic thromboembolic pulmonary hypertension patients. Respir Res..

[26] Mercier O (2017). Abnormal pulmonary endothelial cells may underlie the enigmatic pathogenesis of chronic thromboembolic pulmonary hypertension. J. Heart Lung Transplant..

[27] Osorio Trujillo J (2019). Heterogeneity in lung 18F-FDG uptake in precapillary pulmonary hypertension. Eur. Respir. J..

[28] Shi L, Pan H, Liu Z, Xie J, Han W (2017). Roles of PFKFB3 in cancer. Signal Transduct. Target. Ther..

[29] Li FL (2018). Acetylation accumulates PFKFB3 in cytoplasm to promote glycolysis and protects cells from cisplatin-induced apoptosis. Nat. Commun..

[30] Di R, Yang Z, Xu P, Xu Y (2019). Silencing PDK1 limits hypoxia-induced pulmonary arterial hypertension in mice via the Akt/p70S6K signaling pathway. Exp. Ther. Med..

[31] Paulin R, Michelakis ED (2014). The metabolic theory of pulmonary arterial hypertension. Circ. Res..

[32] Egnatchik RA (2017). Dysfunctional BMPR2 signaling drives an abnormal endothelial requirement for glutamine in pulmonary arterial hypertension. Pulm. Circ..

[33] Alamri A, Burzangi AS, Coats P, Watson DG (2018). Untargeted metabolic profiling cell-based approach of pulmonary artery smooth muscle cells in response to high glucose and the effect of the antioxidant vitamins D and E. Metabolites.

[34] Emini Veseli B, Perrotta P, Van Wielendaele P, Lambeir A-M, Abdali A, Bellosta S, Monaco G, Bultynck G, Martinet W, De Meyer GRY (2020). Small molecule 3PO inhibits glycolysis but does not bind to 6-phosphofructo-2-kinase/fructose-2,6-bisphosphatase-3 (PFKFB3). FEBS Lett..

[35] Batton KA (2018). Sex differences in pulmonary arterial hypertension: role of infection and autoimmunity in the pathogenesis of disease. Biol. Sex Differ..

[36] Hoeper MM, Boucly A, Sitbon O (2018). Age, risk and outcomes in idiopathic pulmonary arterial hypertension. Eur. Respir. J..

[37] Tura-Ceide O, Smolders VFED, Aventin N, Morén C, Guitart-Mampel M, Blanco I, Piccari L, Osorio J, Rodríguez C, Rigol M, Solanes N, Malandrino A, Kurakula K, Goumans MJ, Quax PHA, Peinado VI, Castellà M, Barberà JA (2021). Derivation and characterisation of endothelial cells from patients with chronic thromboembolic pulmonary hypertension. Sci. Rep..

