# Supplementary material for: Metabolic profile in endothelial cells of chronic thromboembolic pulmonary hypertension and pulmonary arterial hypertension
Source: Sci Rep. 2022 Feb 10;12:2283. doi: 10.1038/s41598-022-06238-z (PMC8831561; doi:10.1038/s41598-022-06238-z)
Supplement: Supplementary file 1 — Supplementary Information. [file 41598_2022_6238_MOESM1_ESM.pdf]

Supplementary Material

Metabolic profile in endothelial cells of chronic thromboembolic pulmonary hypertension and pulmonary arterial hypertension.

V.F.E.D. Smolders*[1,2], C. Rodríguez*[1,3], I. Blanco[1,4], R. Szulcek[5,6], Wim Timens[7], L. Piccari[1], Y. Roger[1], X. Hu[1], Constanza Morén [8,9], C. Bonjoch[1], L. Sebastián[1], M. Castellà[10], J. Osorio[1,4], V. I. Peinado[1,4], Harm Jan Bogaard[11], P. H. A. Quax[2], M. Cascante[12,13+], J.A. Barberà[1,4+], O. Tura-Ceide[1,3,4+].

[1] Department of Pulmonary Medicine, Hospital Clínic-Institut d'Investigacions Biomèdiques August Pi i Sunyer (IDIBAPS); University of Barcelona; Barcelona, Spain.[2] Department of Surgery, Leiden University Medical Center, Leiden, The Netherlands.[3]Biomedical Research Institute-IDIBGI, Girona, Spain. [4]Centro de Investigación Biomédica en Red (CIBER) de Enfermedades Respiratorias, Madrid, Spain. [5]Laboratory of in vitro modeling systems of pulmonary diseases, Institute of Physiology, Charité – Universitätsmedizin Berlin, corporate member of Freie Universität Berlin and Humboldt-Universität zu Berlin, Charitéplatz, Berlin, Germany. [6]German Heart Center Berlin, Berlin, Germany. [7]University of Groningen, University Medical Center Groningen, Dept. of Pathology and Medical Biology, Groningen, the Netherlands [8]Laboratory of Muscle Research and Mitochondrial Function, Institut d'Investigacions Biomèdiques August Pi i Sunyer (IDIBAPS), University of Barcelona (UB), Department of Internal Medicine, Hospital Clínic of Barcelona (HCB), Barcelona, Spain. [9]Biomedical Research Networking Centre on Rare Diseases (CIBERER), Madrid, Spain. [10]Department of Cardiovascular Surgery, Institut Clínic Cardiovascular, Hospital Clínic, Barcelona, Spain. [11]Amsterdam UMC, VU University Medical Center (VUmc), Dept. of Pulmonary Diseases, Amsterdam Cardiovascular Sciences (ACS), Amsterdam, the Netherlands. [12]Department of Biochemistry and Molecular Biomedicine and Institute of Biomedicine (IBUB), Faculty of Biology, Universitat de Barcelona, Diagonal 643, 08028 Barcelona, Spain. [13]CIBER of Hepatic and Digestive Diseases (CIBEREHD), Institute of Health Carlos III (ISCIII), 28029 Madrid, Spain.

*Both authors have contributed equally

+Correspondence: olgaturac@gmail.com, martacascante@ub.edu and jbarbera@clinic.cat

Servei de Pneumologia, Hospital Clínic. Villarroel, 170. Barcelona 08036. Spain. Phone 34-93-227-5747; Fax. 34-93-227-5455.

Running title: Metabolic profile in pulmonary hypertension.

Table of contents: Supplementary Tables and figures.

Supplementary table 1. Primers used in this manuscript

Gene name	Forward (5'-3')	Reverse (5'-3')
**G6PD**	CCAAGCCCATCCCCTATATTT	CCACTTGTAGGTGCCCTCAT
**GLS1**	GCTGTGCTCCATTGAAGTGACT	TTGGGCAGAAACCACCATTAG
**GLUD1**	GGGATTCTAACTACCACTTGCTCA	AACTCTGCCGTGGGTACAAT
**GLUT1**	GGTTGTGCCATACTCATGACC	CAGATAGGACATCCAGGGTAGC
**HK2**	TCCCCTGCCACCAGACTA	TGGACTTGAATCCCTTGGTC
**LDHA**	GCAGATTTGGCAGAGAGTATAATG	GACATCATCCTTTATTCCGTAAAGA
**PDHA1**	CCTGACTTTATATGGCGATGG	CTGCCATGTTGTAAGCTTCG
**PDK1**	GGTTACGGGACAGATGCAGT	CGTGGTTGGTGTTGTAATGC
**PFKFB3**	CCTACCTGAAATGCCCTCTTC	GTCCCTTCTTTGCATCCTCTG
**HOXD3**	CGTAAGGATTGCATCGGACT	TCCTAAGCTCGGCTGGATAA
**HOXD8**	TAAACCAGCTTGCTGTGTGC	GTGAGGCTATCGCTTTCCTG
**HOXD9**	CCTGCTCCATTGGTTCCTTA	TCAGAAACATGGGGGACATT

Supplementary table 2. Antibodies used in this manuscript

Name	Dilution	Company	Code	Characteristics	Host	Reactivity	kDa
**HK2**	1/800	santa cruz	sc-374091	monoclonal	mouse	human	100
**LDHA**	1/800	santa cruz	sc-137243	monoclonal	mouse	human	35
**G6PD**	1/10000	abcam	ab993	polyclonal	rabbit	human	59
**PDH-E1α**	1/1000	santa cruz	sc-377092	monoclonal	mouse	human	43
**p-PDH-E1α**	1 µg/mL	abcam	ab92696	polyclonal	rabbit	human	43
**Total OXPHOS Cocktail**	1/250	abcam	ab110413	monoclonal	mouse	human	/[a]
**PDK1**	1/2000	abcam	ab207450	monoclonal	rabbit	human	49
**β-actin**	1/1000	novus biologicals	NB600-503	polyclonal	rabbit	human	37
**Vinculin**	1/10000	abcam	ab129002	monoclonal	rabbit	human	124

[a] This kit contains 5 antibodies. The kDa for each antibody is indicated in figure 4.

Full-length gels represented in Figure 3 in order of appearance.

a)

Figure 3A-B

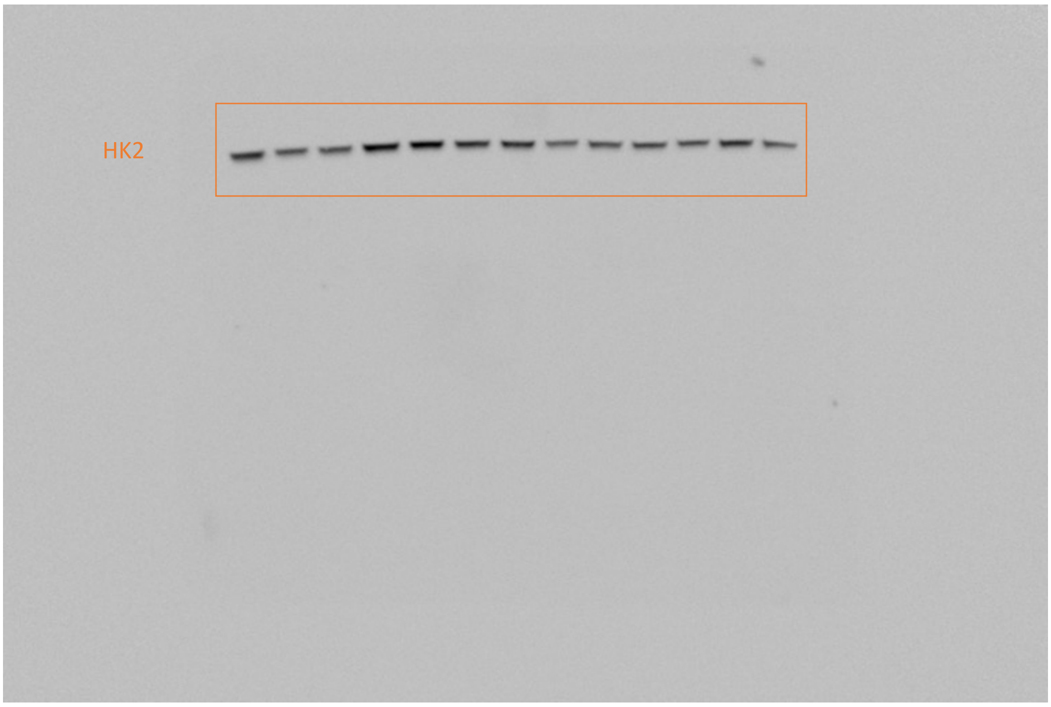

b)

Figure 3A-B

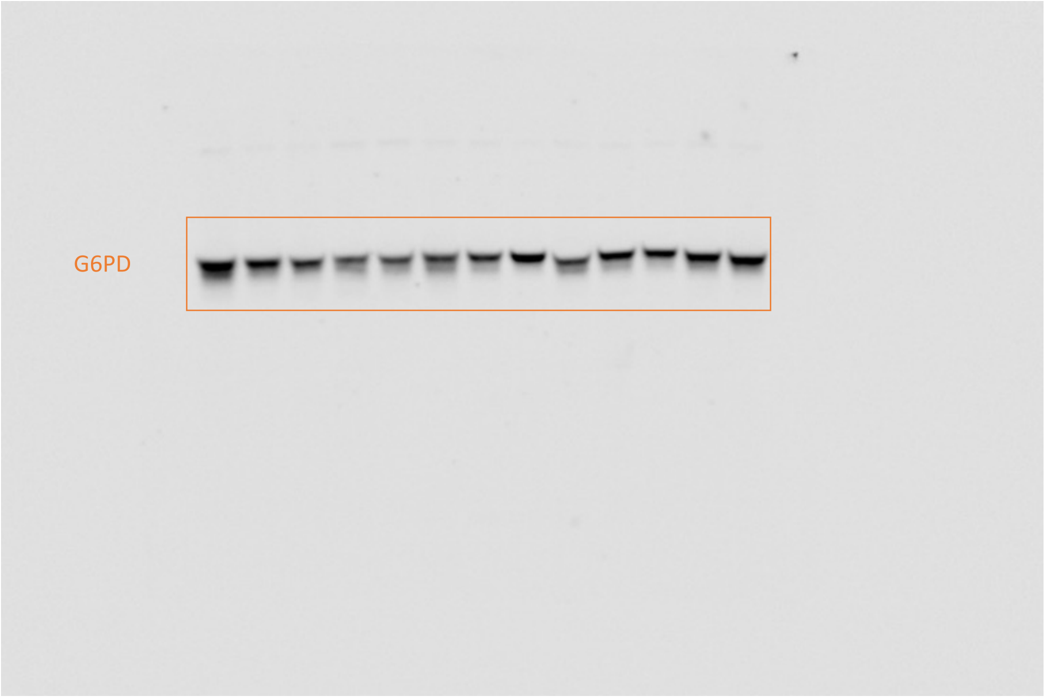

c)

Figure 3A-B

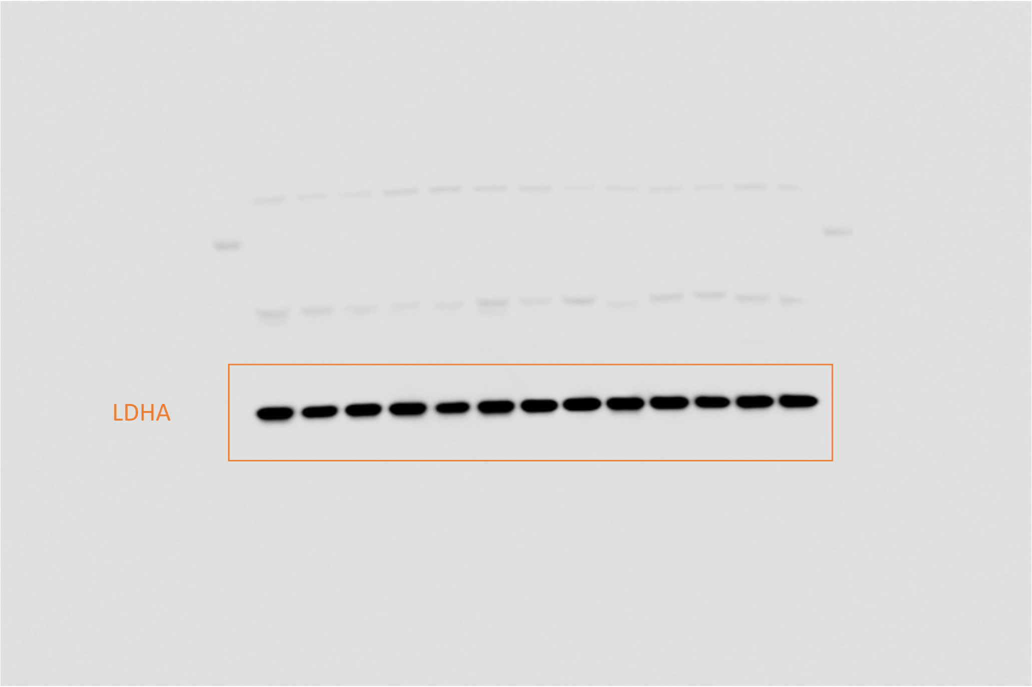

d)

Figure 3A-B

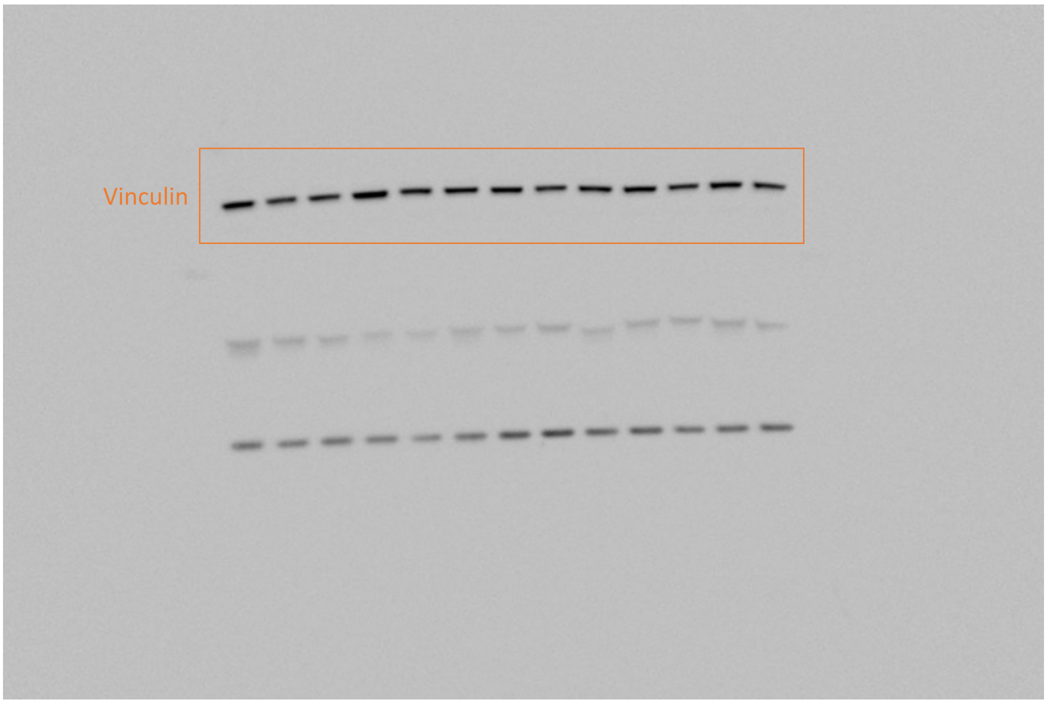

e)

Figure 3C

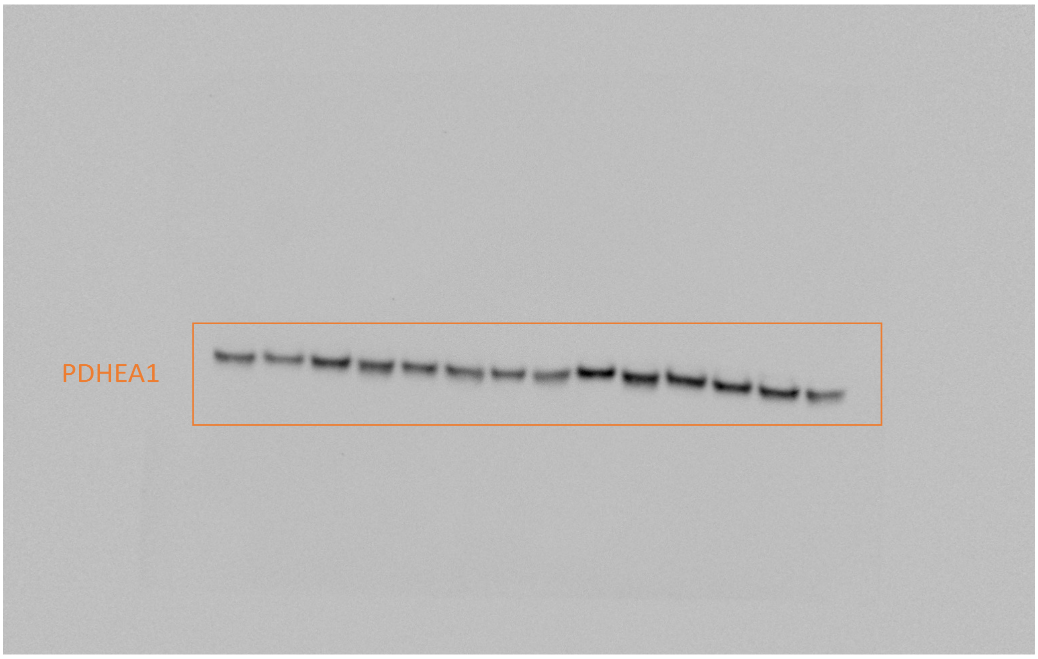

f)

Figure 3C

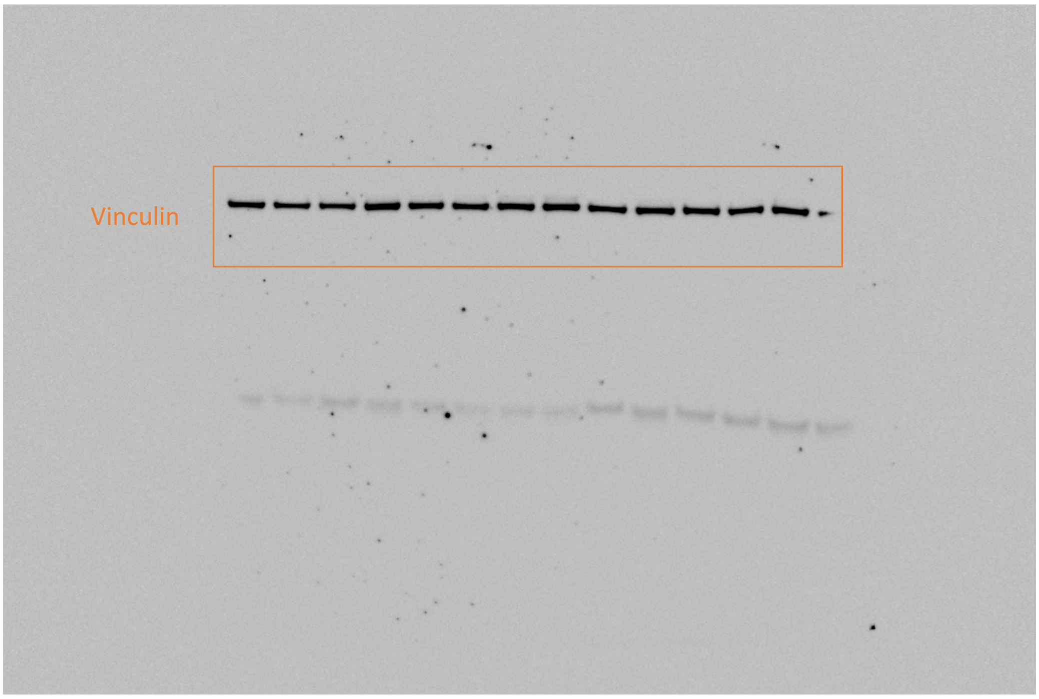

g)

Figure 3C

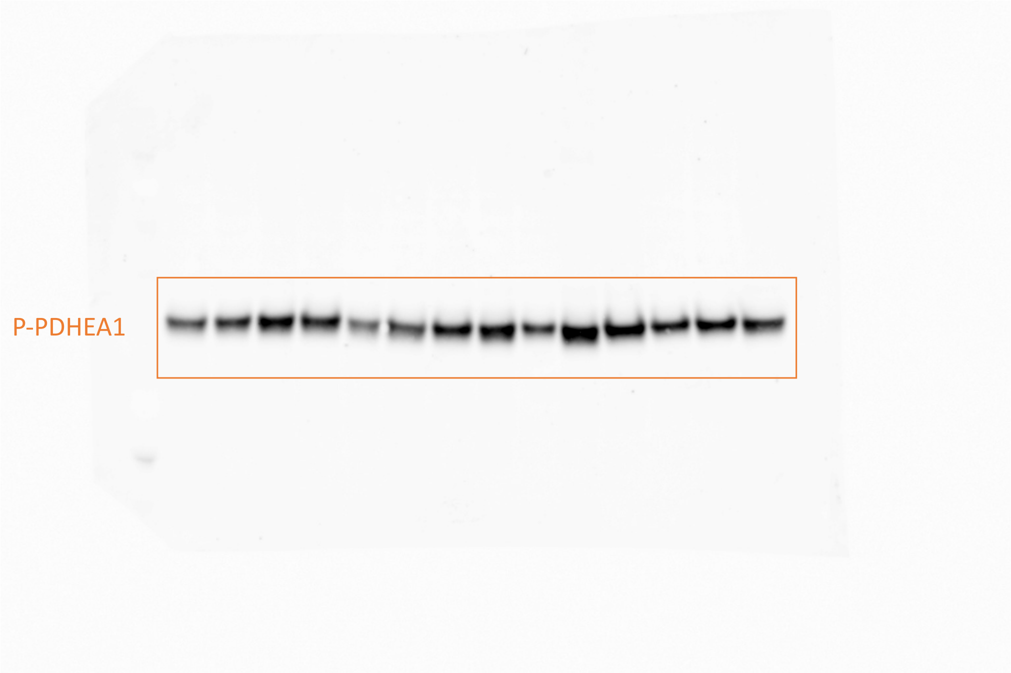

h)

Figure 3C

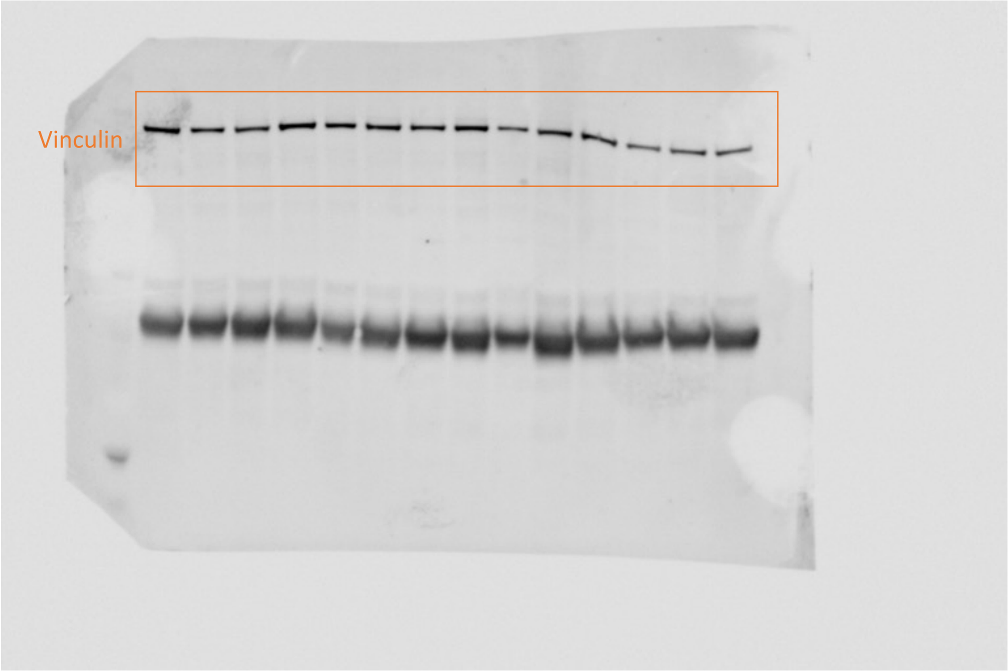

i)

Figure 3C

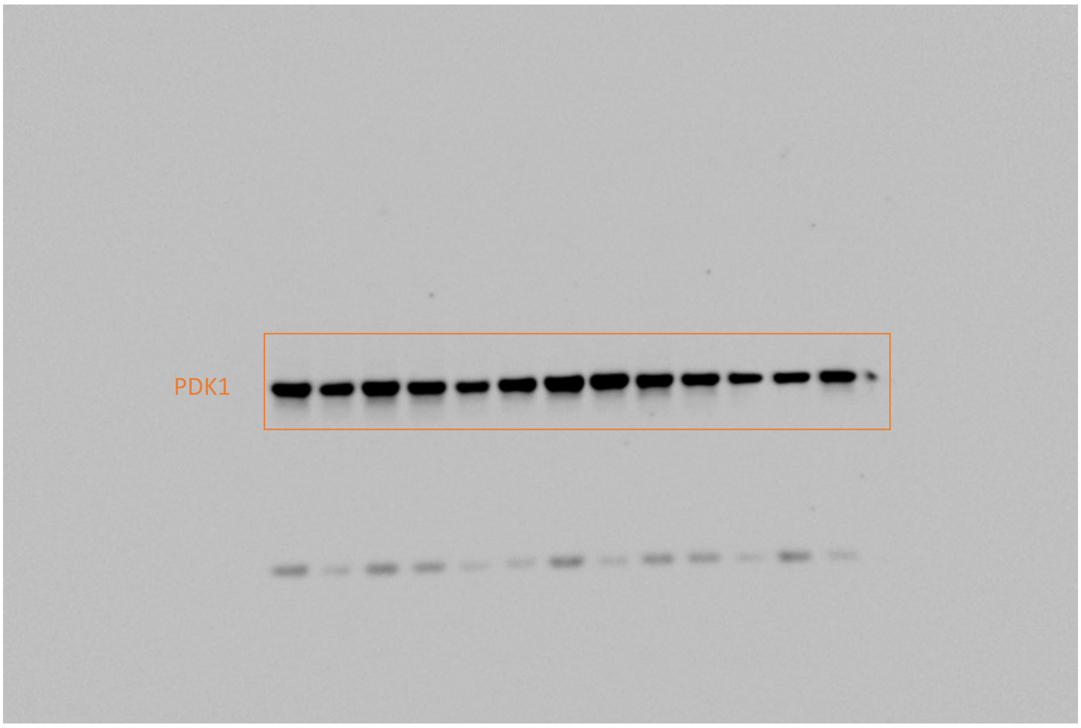

j)

Figure 3C

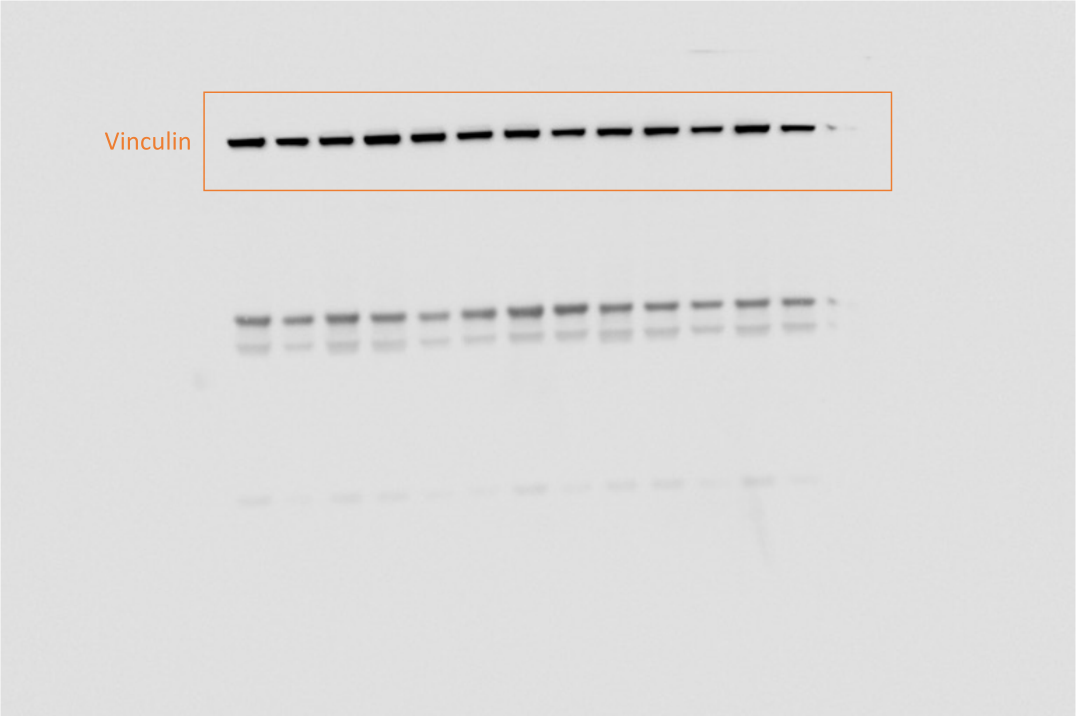

Supplementary Fig. 1. Full-length gels represented in main Figure 3A-C, a) HK2, b) G6PD, c) LDHA, d) Vinculin, e) PDHEA1, f) Vinculin, g) p-PDHEA1, h) Vinculin, i) PDK1 and j) Vinculin.

Full-length gels represented in Figure 4 in order of appearance.

a)

Figure 4

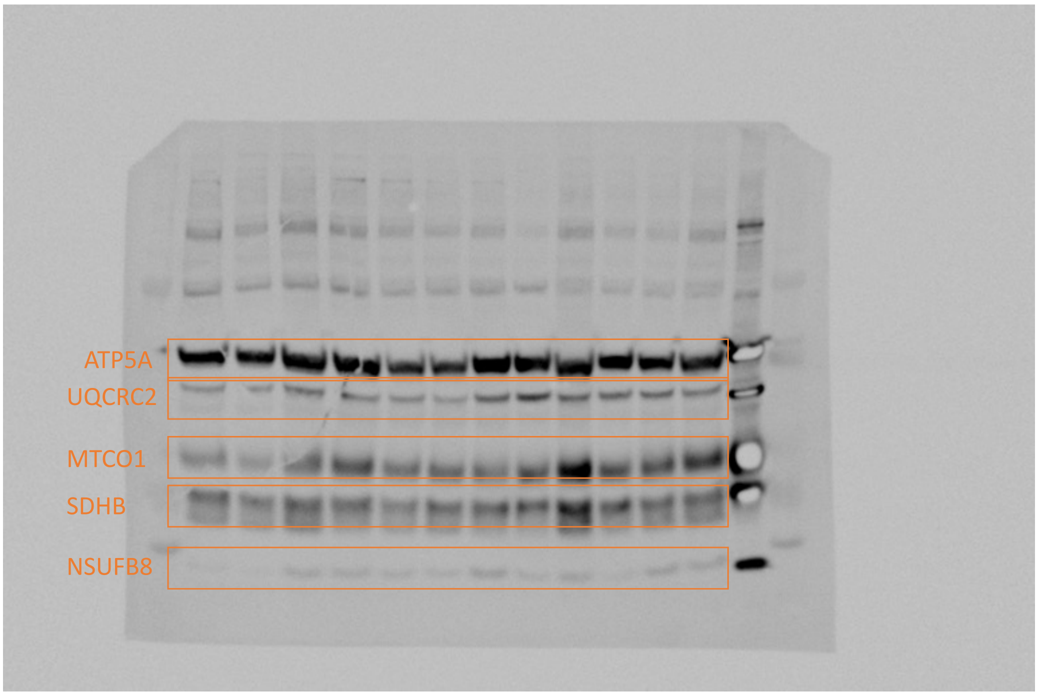

b)

Figure 4

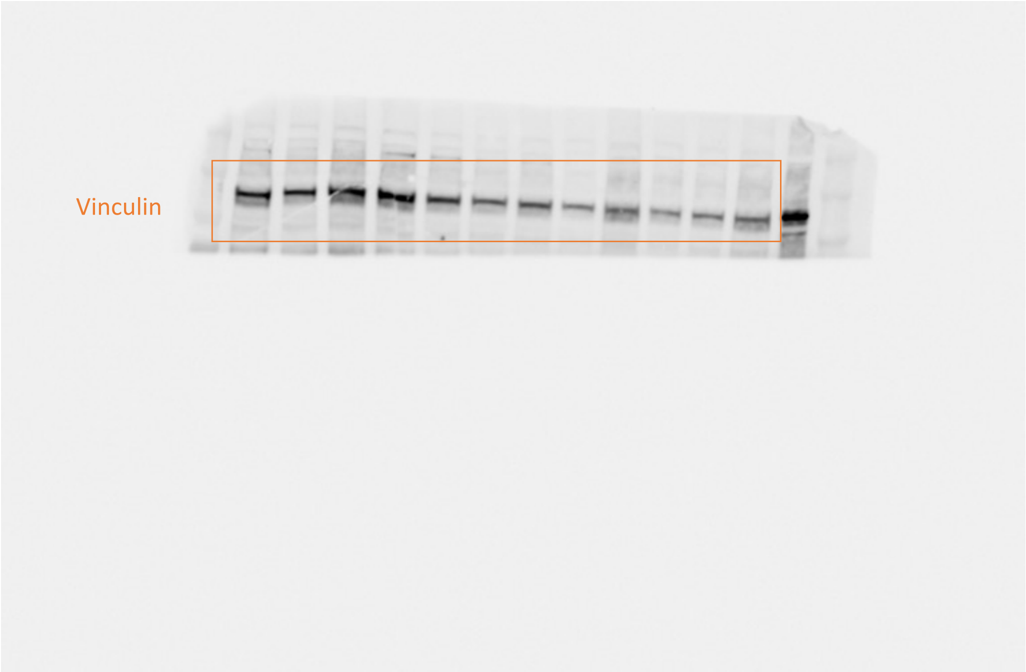

Supplementary Fig. 2. Full-length gels represented in main Figure 4, a) ATP5A, UQCRC2, MTCO1, SDHB and NSUFB8 oxidative phosphorylation associated proteins and b) Vinculin.
